# Two-Dimensional Algal Collection and Assembly by Combining AC-Dielectrophoresis with Fluorescence Detection for Contaminant-Induced Oxidative Stress Sensing

**DOI:** 10.3390/bios5020319

**Published:** 2015-06-15

**Authors:** Coralie Siebman, Orlin D. Velev, Vera I. Slaveykova

**Affiliations:** 1 Environmental Biogeochemistry and Ecotoxicology, Institute F.-A. Forel, Earth and Environmental Science, Faculty of Sciences, University of Geneva, 10 route de Suisse, Versoix CH-1290, Switzerland; E-Mail: coralie.suscillon@unige.ch; 2 Department of Chemical and Biomolecular Engineering, North Carolina State University, Raleigh, NC 27695, USA; E-Mail: odvelev@ncsu.edu

**Keywords:** whole cell assembly, dielectrophoresis, fluorescence, contamination, reactive oxygen species, *Chlamydomonas reinhardtii*

## Abstract

An alternative current (AC) dielectrophoretic lab-on-chip setup was evaluated as a rapid tool of capture and assembly of microalga *Chlamydomonas reinhardtii* in two-dimensional (2D) close-packed arrays. An electric field of 100 V·cm^−1^, 100 Hz applied for 30 min was found optimal to collect and assemble the algae into single-layer structures of closely packed cells without inducing cellular oxidative stress. Combined with oxidative stress specific staining and fluorescence microscopy detection, the capability of using the 2D whole-cell assembly on-chip to follow the reactive oxygen species (ROS) production and oxidative stress during short-term exposure to several environmental contaminants, including mercury, methylmercury, copper, copper oxide nanoparticles (CuO-NPs), and diuron was explored. The results showed significant increase of the cellular ROS when *C. reinhardtii* was exposed to high concentrations of methylmercury, CuO-NPs, and 10^−5^ M Cu. Overall, this study demonstrates the potential of combining AC-dielectrophoretically assembled two-dimensional algal structures with cell metabolic analysis using fluorescence staining, as a rapid analytical tool for probing the effect of contaminants in highly impacted environment.

## 1. Introduction

The generation of the reactive oxygen species (ROS) and the oxidative stress is currently one of the most promising paradigms to assess and compare the toxicity of different contaminants in the environment [1,2,3,4,5]. ROS, comprising both free oxygen radicals and non-radicals derivatives at low to moderate levels, are involved in essential biological functions such as cellular signaling or cell proliferation [5]. However, overproduction of ROS, surpassing a cell’s antioxidant capacity, can lead to oxidative stress and consequent DNA, lipid, or protein damages, inducing possible cell death [5]. Indeed, environmental contaminants, such as toxic metals, synthetic organic substances, and engineered nanomaterials have been shown to influence cellular metabolic processes by enhancing the intracellular generation of ROS in exposed organisms [2,6,7,8,9,10,11]. In parallel to the system biology approach [12], a large spectrum of measurement techniques are being developed to provide valuable information on the biological production of ROS and oxidative stress. The advantages and disadvantages of the most widely used approaches for ROS and oxidative stress determination are discussed in a few recent review papers: EPR spin trapping for radical detection [13], electrochemical (mainly enzymatic) biosensors [14], bioassays combined with various ROS-specific or unspecific fluorescence probes [15] or antioxidant capacity [16]. Optical biosensors, based of plasmon resonance detection for highly sensitive real-time measurement of extracellular H_2_O_2_ released from cells, were also developed [17,18,19].

Whole-cell biosensors using microalgae as biological component allow rapid and sensitive screening of contaminants in environmental samples and could become viable alternative of the classical ecotoxicity bioassays [20,21,22]. However, reproducible immobilization of the living cells in facile assembly format for biosensing is still challenging and their viability is difficult to maintain [20,23,24]. Numerous immobilization techniques including encapsulation [24,25,26], covalent binding [22,27,28], or adsorption to surfaces [22,29] have revealed several drawbacks, such as the possible interference with an optical detection [24] or the possible toxicity of the immobilization chemicals limiting their environmental application [28]. In this context, recent advances in the field of electrokinetic single cell handling offer an opportunity for rapid on-chip cell concentration and capturing [30,31]. Such techniques, and especially the use of alternative current (AC) dielectrophoresis (DEP), enable researchers to separate, trap, and focus live cells suspended in a fluid medium [32,33]. AC-DEP has already been deployed to isolate damaged cells in biological and medical sciences with numerous target cells such as blood erythrocytes and cancer cells [30,34,35]. Nonetheless, few studies deal with DEP handling of cells for biosensing in natural environment as reviewed in [23]. The potential of DEP as a tool for chaining of live green microalga *Chlamydomonas reinhardtii* in ambient water using coplanar electrodes was recently demonstrated [36]. The collection of alga *Selenastrum capricornutum* exposed to copper on the two needles electrode was also reported [37]. To our knowledge no studies combining the key technique of DEP cell manipulation with fluorescent probes with environmentally relevant microorganisms trapped by four point needle electrodes and detection of ROS production have been reported.

The current study combines on-a-chip concentration and capture in two-dimensional (2D) assembly by positive AC-DEP of alga *C. reinhardtii*, using four point needle electrodes, with fluorescence probe detection with the aim of characterizing the cellular ROS production during short-term exposure to various contaminants. The influence of the AC electric field intensity, frequency, and duration on the efficiency of the dielectrophoretic assembly of *C. reinhardtii* was studied. The possible effect of the DEP and electrode material on the cellular oxidative status was also characterized. Next, the 2D-assemblies combined with ROS-probe and fluorescence microscopy were used to follow the evolution of the oxidative status of *C. reinhardtii* exposed to mercury and methylmercury, copper and copper oxide nanoparticles, and diuron. The observations obtained with the 2D-assemblies were validated by flow cytometry.

## 2. Experimental Section

### 2.1. Algal Cell Cultures and Test Media

Unicellular green alga *Chlamydomonas reinhardtii* (CPCC 11, Canadian Phycological Culture Centre, Waterloo, Canada) was cultured at 20 °C under rotary shaking at 115 rpm and continuous illumination of 6000 lux (INFORS HT, Basel, Switzerland) in a four times diluted Tris-Acetate-Phosphate medium (Sigma-Aldrich, Buchs, Switzerland) to the mid-exponential growth phase. The cells were collected by centrifugation at 3000 rpm for 5 min (Omnifuge 2.0 RS, Heraeus Sepatech GmbH, Osterode/Harz, Germany). The supernatant was removed and the cells were suspended in the test medium to reach a final concentration of 5 × 10^6^ cells·mL^−1^. The test medium contained 10^−4^ M 3-(N-Morpholino)propanesulfonic acid (MOPS) (Sigma-Aldrich, Buchs, Switzerland) in the absence and presence of various contaminants. Stock standard solutions of 1 mg·L^−1^ methylmercury (MeHg), 1 mg·L^−1^ mercury (Hg) in 12% nitric acid, 0.1 M copper sulfate (Cu), and diuron were obtained from Sigma-Aldrich. Stock solution of 10^−3^ M diuron was filtered through 0.45 µm-pore size filters (Millipore, Billerica, USA). Copper oxide nanoparticles of 99.1% purity were obtained from Nanostructured and Amorphous Materials (Houston, TX, USA). The stock dispersion of 2 g·L^−1^ was prepared in MilliQ water and sonicated at 130 W, 20 kHz for 1 min prior use (Sonics Vibra Cell, Sonic and Materials, Newtown, PA, USA).

The average hydrodynamic size and zeta potential of CuO-NPs in 10^−4^ M MOPS determined by Zetasizer Nano-ZS (Malvern, Renens, Switzerland) were 241 ± 13 nm and −22.2 ± 0.6 mV, respectively. CuO-NPs suspensions contained less than 10% dissolved Cu, determined by inductively coupled plasma mass spectrometry after dialysis (1000 Da cut-off of the membrane).

### 2.2. DEP Experimental Set-Up

**Figure 1:**
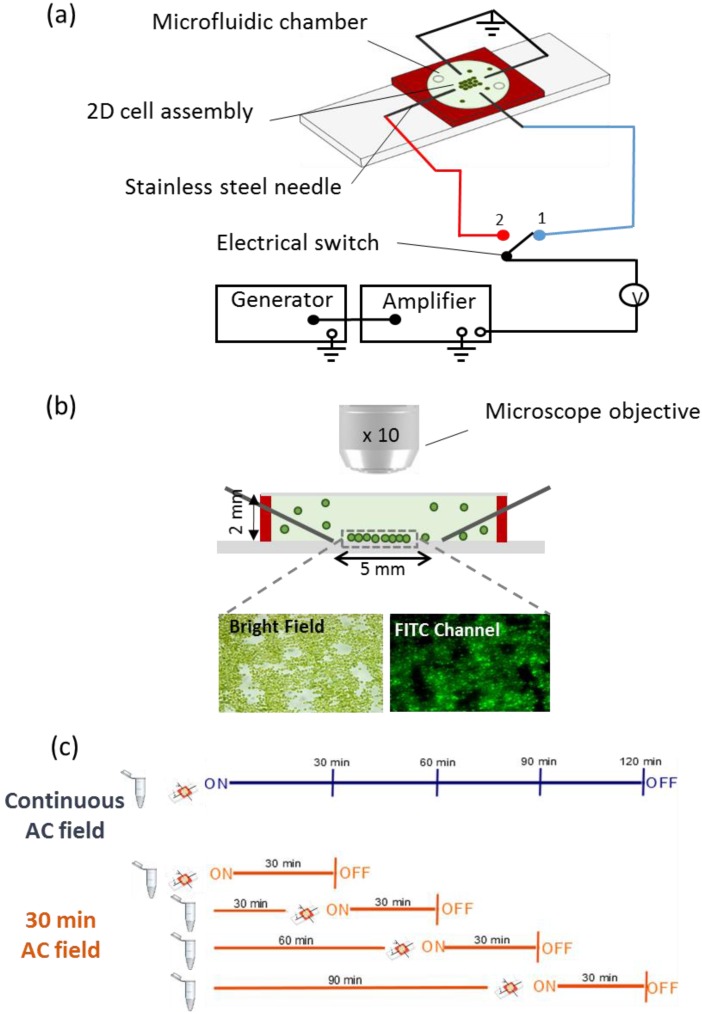
(**a**) Schematics of the experimental set-up. The four orthogonally stainless steel needle electrodes were connected in pairs to the generator and amplifier. The electric field was applied to one pair of electrodes and was switched to the other pair every 2 min allowing the formation of 2D-cell assembly. The cells and media are confined in a microfluidic chamber of silicone rubber (red) with optically transparent top; (**b**) Transverse section of the microfluidic chamber. The 2D-assembly of cells formed in the 5 mm-gap of the four point needle electrodes is observed by microscope using bright field and fluorescence channel FITC. The chamber and the cell sizes are not to scale; (**c**) Experimental plan used to test the effect of the collection time on the cell’s oxidative status.

DEP assembly experiments were performed with four-point needle electrodes separated by a 5 mm-gap and orthogonally arranged around a transparent top microfluidic chamber of 2 mm-height (HybriWell Incubation Chamber, BioCat GmbH, Heidelberg, Germany) (Figure 1a).

The transparent microfluidic chamber and stainless steel needles were sealed on a 25 × 75 mm microscope glass slide (Fisher Scientific, Pittsburgh, PA, USA). Each pair of needles was connected to a waveform generator (33220A Function, Agilent Technologies, Morges, Switzerland) through a voltage amplifier (2340 Single Channel Amplifier, Tegam, Geneva, OH, USA). Four point needle electrode configuration was chosen because of its capacity to collect and assemble yeast cells in planar 2D-structures while allowing for easy microscope analysis without out-of focus cell background [38]. Moreover, the electric field is assumed to be homogeneous in the middle of the gap where the cells are forming 2D structures [39] and the *C. reinhardtii* are subjected to a uniform benign field.

*C. reinhardtii* suspension in the absence and presence of contaminants was injected into the chamber where the AC-field was applied in two perpendicular directions. The AC-field was first applied in one direction for 2 min, and then switched to the second pair of needles for 2 min. The switching cycle was repeated several times for a total duration of 30 min, if not otherwise specified.

### 2.3. DEP Parameters Optimization

The electrical field frequency, voltage and duration, and the type of needle material were systematically tested to find the most appropriate combination of these DEP parameters allowing the formation of close-packed cellular assemblies, without significant effect on algal oxidative status. AC-field voltages of 60 V·cm^−1^, 80 V·cm^−1^, and 100 V·cm^−1^ were applied. At fixed voltage the frequency was varied from 100 to 1000 Hz. The effect of collection times from 30 min to 120 min on 2D-assembly formation was also explored. Electrodes made from stainless steel and brass needles were compared. After each experiment the microfluidic chamber was thoroughly flushed 3 times with MilliQ water to eliminate possible residues from the previous runs.

### 2.4. Effect of AC-Field on ROS Production by Algal Cells

To determine if AC-field could affect the algal cells oxidative status, the change of fluorescence intensity of the 2D-assemblies in the experimental setup was detected with and without AC-field application. To this end CellROX^®^ Green (Life Technologies Europe B.V, Zug, Switzerland) intracellular oxidative stress indicator, designed to probe cellular ROS production, was used for staining the cells trapped in 2D-assembly. This new generation stain was chosen because of its high stability and lack of interferences with the algal autofluorescence. A volume of 2 µL of 25 mM stock solution of CellROX^®^ Green was added per 1 mL of algal suspension prior to application of AC-field. The mixture was injected into the microfluidic chamber and images were taken by fluorescence microscopy every 30 min. Images corresponding to (i) no AC-field applied, (ii) 120 min continuous AC-field duration, and (iii) 30 min AC-field duration every 30 min until 120 min (Figure 1c) were compared.

To validate the results obtained by the 2D-assembly approach, the possible effect of the AC-field on ROS generation was investigated in parallel by a combination of CellROX^®^ Green stain with flow cytometry (FCM). FCM analyses were performed using a BD Accuri C6 flow cytometer (BD Biosciences, San Jose, CA, USA) with an Accuri CSampler (BD Biosciences, San Jose, CA, USA). The data were acquired and analyzed using the BD Accuri C6 Software 264.15 (BD Biosciences, San Jose, CA, USA). The number of counted cells and CellROX^®^ green fluorescence from green channel (530 ± 15 nm) when excited with 488 nm argon laser. Data were collected to 10,000 events for each sample. Positive control was obtained by adding 8 µL of 100 mM cumene hydroperoxide stock solution (Life Technologies Europe B.V, Zug, Switzerland) into 1 mL algal suspension intermixed with 2 µL of 25 mM stock solution of CellROX^®^ Green.

### 2.5. Sensing of Oxidative Stress during Short-Term Contaminant Exposure

To explore the potential of different contaminants to induce cellular oxidative stress, *C. reinhardtii* was exposed for 2 h to: 10^−9^ M and 10^−7^ M MeHg, 10^−8^ M and 10^−7^ M Hg, 10^−5^ M, 5 × 10^−6^ M and 10^−6^ M Cu, 50 and 10 mg·L^−1^ CuO-NPs and 10^−7^ M and 10^−6^ M diuron. These concentrations of Cu, Hg, CuO-NPs and diuron were chosen based on the previous results for lipid peroxidation in *C. reinhardtii* obtained by FCM [40]. The algal suspensions were exposed to each contaminant for 30, 60, 90 and 120 min. 2 µL of 25 mM CellROX^®^ Green were added to the suspension before the injection into the microfluidic chamber and AC-field was applied for 30 min. Algae following the same treatment in the absence of contaminant were used as negative control. Each exposure experiment was repeated 3 times.

### 2.6. Fluorescence Microscopy and Image Analysis

The DEP-induced formation of the 2D close-packed cell arrays was observed with fluorescence microscopy (BX61, OLYMPUS, Volketswil, Switzerland) using a digital camera (XC30, OLYMPUS Volketswil, Switzerland) and the Cellsens software (Cellsens dimension OLYMPUS Volketswil, Switzerland). For each experiment, images of bright field and FITC channel were collected at 30, 60, 90, and 120 min.

The efficiency of the 2D-assembly process was characterized by the percentage of microfluidic chamber surface covered by cells. In CellROX^®^ cellular fluorescence detection, the out-of-focus background fluorescence was subtracted from on-focus cellular fluorescence intensity by using Equation (1) [41]:

(1) $$CF_{obj}=\;\overset{i=N_{obj}}{\underset{i=1}{\sum }}F_{obj\;i}-N_{obj}\;\frac{\sum_{J=1}^{J=N_{obj}}F_{bkg\;j}}{N_{bkg}}$$

where *CF* is the corrected fluorescence; *F* is the fluorescence intensity measured at each pixel in the object and in the background respectively *i* and *j*, *obj* is the object of interest defined as the 2D cellular assembly in this study, *bkg* is the background and *N* is the number of pixel in the object of interest or the background [41]. The value of *CF* was then normalized by the area of the object to obtain the mean *CF* value per pixel. The digital images were processed with Image*J* [42]. The calculation was performed on 8-bit images with Image*J*.

### 2.7. Data Processing

Statistical differences within CellROX^®^ response under different treatments (e.g., DEP parameters, contaminants) were evaluated using the Student-Neuman-Keuls test for multiple comparison and one-way ANOVA in Sigma Plot 11 (Systat Software Inc., San Jose, CA, USA).

## 3. Results and Discussion

### 3.1. Formation of Algal 2D Close-Packed Structures

**Figure 2:**
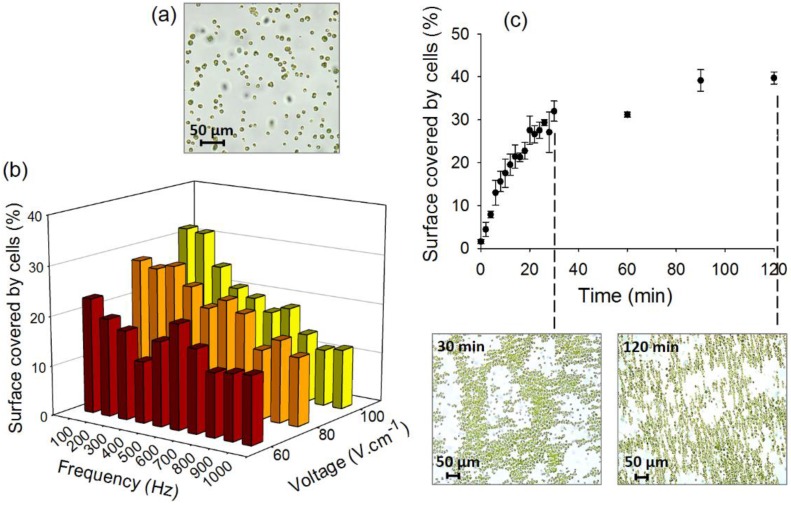
(**a**) Bright-field image of the algal cell suspensions in the microfluidic chamber with no AC-field applied; (**b**) Effect of field intensity and frequency; and (**c**) duration on the percentage of the surface of the chamber covered by cells. The bright-field images represent the 2D-assembly of *C. reinhardtii* obtained at combination of 100 V·cm**^−^**^1^, 100 Hz after 30 and 120 min duration using four-point stainless steel needle electrodes. Initial concentration of algae in suspensions: 5 × 10^6^ cells·mL**^−^**^1^.

The key role of DEP on the process of cell assembly was confirmed first by comparing results obtained in the setup without AC-field and with AC-field applied for 30 min, as well as the effect of AC-field intensity and frequency (Figure 2). The application of AC electric field allowed the formation of a closely packed cell structure, defined further as 2D-assembly without any 3D-stacking, covering 31.9% ± 3.7% of the microchamber surface. This is a consequence of the 2D-algal cell assembly being a two stage process. 1D-cell chains are first formed in the direction of the applied electric field due to the attractive axial dipolar interactions. The AC field was then switched to the other electrode pair to let the cells realign along the perpendicular direction. These membrane-like structures disassemble when the electric field is switched off. When no AC-field was applied, 30 min after injection, highly dispersed cells covering 14.8% ± 1.3% of the microfluidic chamber surface were observed.

As DEP using four point needle electrodes with *C. reinhardtii* had never been explored, the efficiency of formation of the 2D-compact structures was first characterized for different AC-field voltages, frequencies and durations. An increase of the field intensity, establishing the major DEP driving force, from 60 to 100 V·cm^−1^ led to an increase of the surface covered by algal cells from 23.1% ± 2.5% to 32.0% ± 2.4% at 100 Hz (Figure 2b). The tested fields were limited to 100 V·cm^−1^ because of the formation of bubbles and flows near the electrodes at low frequency and higher voltages. The flows could be explained by an AC-electroosmotic (ACEO) phenomena, where the large field intensities in the chamber lead to macroscopic fluid flows that could disrupt the cellular array. ACEO flows at high voltages and low frequencies have already been previously reported for algal cells [36]. The above results are consistent with previous studies where the chaining of yeast, bacterial, and algal cells increased with the intensity of the electric field [36,39,43]. Indeed, the increase of the field at 100 Hz resulted in more efficient chaining of *C. reinhardtii* in ambient water [36] and of baker’s yeast cells [39]. Similarly, the increase of an AC-field intensity from 67 V·cm^−1^ to 84 V·cm^−1^ has improved the capture efficiency from 90% to 99% for bacteria *E. coli*, *Salmonella* and *Pseudomonas* sp. between two planar gold electrodes [43] while a decrease from 5 to 2 Vpp (peak to peak voltage) induced respectively the release of *Cryptosporidium muris* and *Giardia lambia* trapped between interdigitated electrodes [44]. DNA trapping on interdigitated electrodes has also been shown to be more effective with an increase of the voltage from 0.5 to 2.5 Vpp [45].

An increase of the frequency of AC-field from 100 Hz to 1000 Hz induced a significant decrease of the percentage of cell surface coverage for the tested voltages, more pronounced at higher voltages. For example at 100 V·cm^−1^ the cell surface coverage decreased from about 32% to 12% when the frequency was increased tenfold (Figure 2b). Similarly, DNA trapping using gold interdigitated electrodes has been more efficient at frequencies lower than 100 Hz [45] and formation of longer chains of yeasts cells between coplanar electrodes has been observed at frequencies below 200 Hz [39]. However, another study demonstrated that a decrease of the frequency from 10 MHz to 100 kHz caused the yeast to be repelled from curved gold microelectrodes [46]. Interestingly, the surface coverage that we measured displays a small peak around 600 Hz for 60 and 80 V·cm^−1^, which may be a result of the increased complex multi-component cell polarizability at low frequencies [31,32], the precise measurement of which, however, is outside the scope of the present paper.

The effect of collection time duration of the AC-field on the efficiency of 2D-assembly formation was also characterized. Cell collection time of 30 min was necessary to obtain about 32% of surface coverage (Figure 2c). Increasing the time from 30 min to 120 min allowed an increase of 1.2 times of the percentage of cell surface coverage (from 32.0% ± 2.4% to 39.7% ± 1.4%). However, further increase of the duration from 90 min and 120 min did not show a significant effect on the cell assembly efficiency. This could be explained by depletion of the free cells following the intense assembly, as also noticed earlier with chaining of green algae using coplanar gold electrodes [36]. In addition, a Joule heating effect and related temperature gradient (and macroscopic fluid flows) could lead to disruption of the cellular assembly. After comparing the data for all tested parameters, the combination of 100 V·cm^−1^ field intensity, 100 Hz frequency and 120 min duration was defined as the optimal condition to obtain close-packed cell assembly.

### 3.2. Effect of Electrode Material and DEP Parameters on Cellular Oxidative Stress

**Figure 3:**
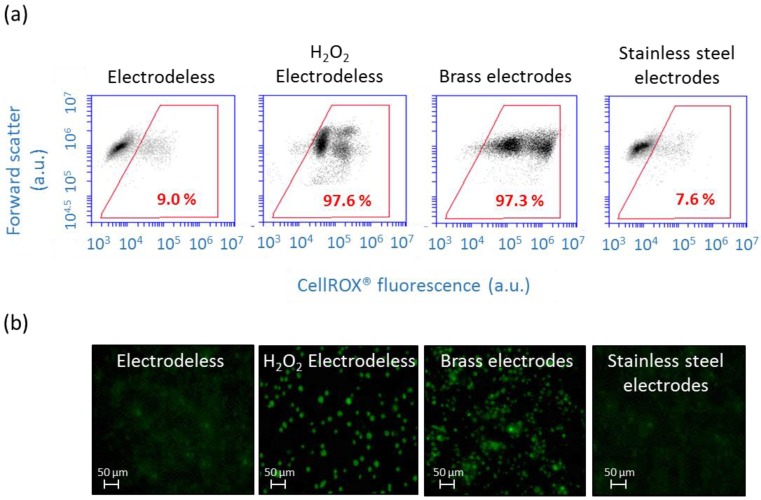
Effect of electrode material on ROS production of *C. reinhardtii* after 30 min with no AC-field. (**a**) Two dimensional dot plots represent forward scatter (FSC) *versus* fluorescence green signal of CellROX^®^ Green obtained by flow cytometry. Percentages in red indicate the proportion of stressed cells stained with CellROX^®^ Green; (**b**) Micrographs obtained with fluorescence microscopy represent algae observed with the fluorescent FITC filter allowing the detection of CellROX^®^ Green.

For the correct functioning of the biosensor the electrode and microfluidic chamber materials should not induce oxidative stress in algae. Therefore, the generation of the cellular ROS in algae was compared in the devices with brass needles and stainless steel electrodes (Figure 3). In the set up with stainless steel electrodes, the percentage of cells with enhanced ROS was comparable to the results for cell in the chamber when no AC-field was applied, as demonstrated by FCM. By contrast, 97.3% of the cells were CellROX^®^ positive for 30 min with no AC-field and brass electrodes, showing that these electrodes are unsuitable for biosensing purposes. For comparison, after 30 min into the chamber without electrodes, only 9% of the algal cells showed an increase in ROS production using FCM and no bright green fluorescence was observed by microscopy in the absence of AC-field (Figure 3a). In the positive control obtained by exposing the cells to 5 mM of H_2_O_2_ for 1 h, increase of ROS production was induced in 97.6% of the population and all cells showed a bright green fluorescence under fluorescence microscopy. In agreement with FCM results, fluorescence microscopy images revealed cellular assembly bright green fluorescence in the brass electrode setup only, but no signal in the stainless steel electrode setup (Figure 3b). The high oxidative stress observed in the setup with brass electrodes is probably due to the release of Cu and Zn when the suspension of cells was injected to the chamber. Thus, the stainless steel electrodes were best fit for AC-field cell immobilization for biosensing purposes. No effect of the chamber material on the cellular ROS was found by comparing the % of the CellROX^®^ stained cells injected in the microfluidic chamber and in native algal suspension (Figure 3a).

Further important issue in the development of oxidative stress whole cell biosensor is that the DEP cell manipulation should not induce measurable oxidative stress response. Existing literature report no effect of AC-field on the viability of yeast [39], endothelial cell [47] and lipid peroxidation of green alga *C. reinhardtii* [36], however, no such information is available for the electrode set-up used in the present study. The lack of the significant oxidative stress changes induced by positive DEP in the present set-up was confirmed by FCM measurements.

**Figure 4:**
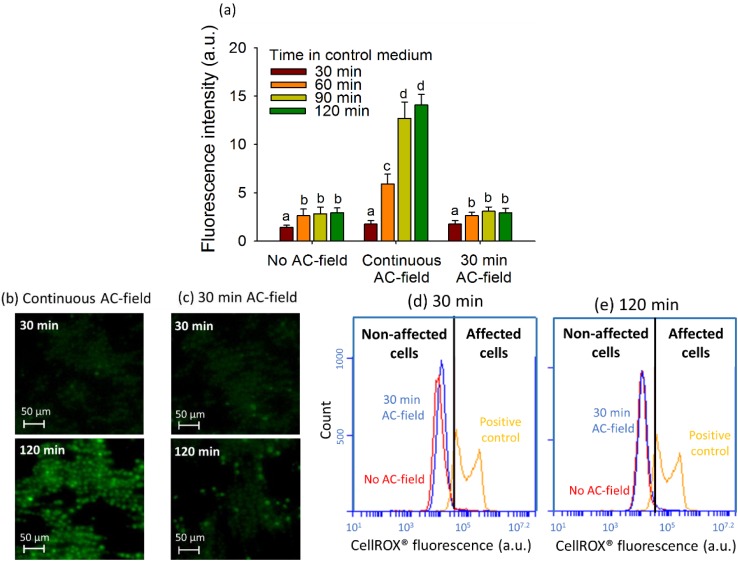
Effect of the AC-field duration on fluorescence intensity of 2D cell assemblies. (**a**) Fluorescence intensity corresponding to the mean corrected CellROX^®^ fluorescent intensity per pixel calculated from the fluorescent images obtained in FITC channel for different modes of the AC-field application. Different letters (from (**a**) to (**d**)) indicate the existence or not of the statistically significant difference and were obtained by Student-Newman-Keuls test with *p* < 0.05; Microscopy images of CellROX^®^ stained 2D-assembly with continuous AC-field (**b**) and AC-field applied for 30 min (**c**); FCM cytograms for CellROX^®^ Green stained cells with no AC-field and AC-field applied for 30 min for exposure in the control medium for 30 min (**d**) and 120 min (**e**). The initial concentration of algae in suspensions was 5 × 10^6^ cells·mL^−1^.

No shift in the fluorescence intensity of CellROX^®^ Green stained cells between AC-field of 100 V·cm^−1^ applied for 30 min and 120 min and no AC-field was observed in the flow cytograms (Figure 4d,e). For a cell collection duration of 30 min, the combination of field intensity/frequency of 100 V·cm^−1^/100 Hz has no significant effect on the *CF* of the CellROX^®^ positive cells indicating no enhancement of intracellular ROS (Figure 4a,c). The *CF* values were comparable with those determined in the control measurement with AC-field applied, where *CF* increased from 1.42 ± 0.22 for 30 min to 2.94 ± 0.49 for 120 min. However for the same field intensity/frequency combination, longer DEP duration resulted in *CF* increase from 1.75 ± 0.38 for 30 min to 14.1 ± 1.1 for 120 min, suggesting significant increase in the algal ROS generation upon longer exposure and collection times. Such increase of ROS generation in the algal cells could be due to the release of heat from the continuous electric field application. Moreover, the electric field could induce undesired motion of internal organelles through the outer cell membrane as shown with the chloroplasts of *Eremosphaera viridis* [48]. Similarly, DEP treatment of yeast cells for longer than 4 h reduced significantly the number of viable cells by 56.8%–89.7% [49]. Positive DEP has only been suitable for short time (<10 min) trapping of genetically modified *E. coli* [50]. Overall, the results demonstrate the importance of the careful selection of the AC-field duration to avoid detrimental long-term field effects on cell oxidative status.

### 3.3. Determination of Cu Induced Oxidative Stress by 2D-Assembly

To explore the capabilities of the 2D-assembly to determine the contaminant induced oxidative stress, the cells of *C. reinhardtii* where exposed to increasing Cu concentrations, stained with CellROX^®^ Green and observed with fluorescence microscopy. Cu was chosen since it is known to induce oxidative stress in algae [2,40]. Indeed, high concentrations of dissolved Cu induced ROS accumulation and oxidative stress in *C. reinhardtii* [6,40], *Pseudokirchneriella subcapitata* and *Chlorella vulgaris* [51].

**Figure 5:**
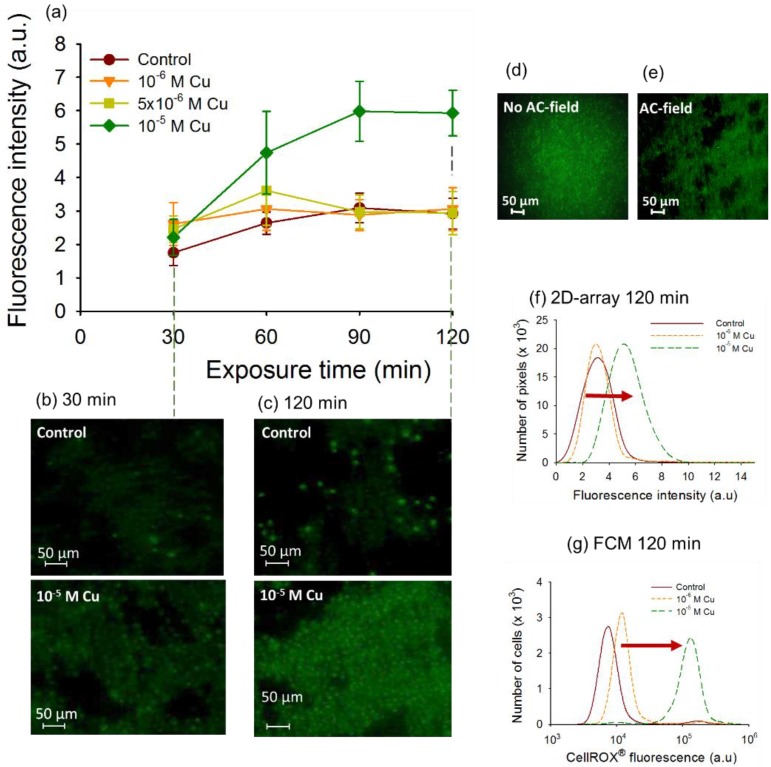
Data for Cu-induced oxidative stress in 2D-assemblies of *C. reinhardtii.* (**a**) Time course of the mean corrected CellROX^®^ Green fluorescent intensity (*CF*) per pixel of cells assembled in 2D-arrays by DEP exposed to increasing Cu concentrations; (**b**) Microscopy images of CellROX^®^ stained 2D-assemblies in the absence and presence of Cu for 30 (**b**) and (**c**) 120 min of exposure; Fluorescence images of CellROX^®^ stained cells after 30 min of exposure to 10^−5^ M Cu (**d**) no AC-field applied and (**e**) AC-field of 100 V·cm^−1^ and 100 Hz; (**f**) Number of pixels obtained with the 2D-assembly *versus* corrected fluorescence of CellROX^®^ Green; (**g**) FCM cytograms for CellROX^®^ Green for 120 min of exposure. Conditions are the same as for 2D-assembly measurements.

No oxidative stress was found for exposure of *C. reinhardtti* to Cu concentrations below 10^−5^ M, where the *CF* values were comparable with those for unexposed controls (Figure 5a). The above observation suggests that the pro-oxidants in the cell can be efficiently balanced by the antioxidant system of the cell. This suggestion is consistent with the enhanced level of expression of genes involved in the enzymatic antioxidant response in algae exposed to comparable concentrations of Cu for 30 and 120 min [10]. 30 min exposure to 10^−5^ M Cu resulted with no oxidative stress induction in both 2D-assemblies and FCM measurements, which is in agreement with up-regulation of antioxidant genes. Significant increase of *CF* of the CellROX^®^ stained cell was observed when 2D-cell arrays were exposed to 10^−5^ M Cu (Figure 5a–c) for times longer than 60 min, suggesting that the ROS generation overwhelms the antioxidant capacity of the cells at this high exposure concentrations. Indeed no further enhancement of the antioxidant gene expression was determined at 120 min exposure [10]. Furthermore, comparison of the fluorescence of the CellROX^®^ stained cells with and without AC-field revealed high fluorescence background around the cells, likely caused by cells sedimentation and dispersion in different heights of the chamber when no AC-field was applied (Figure 5d). Such strongly interfering background fluorescence interference was not observed when cells were assembled in 2D-array. Thus, DEP cell assembly was proved essential in collecting the microalgae and arranging them into a 2D-arrays. Additionally, the formation of 2D single layer close-packed structures without 3D-stacking facilitated fluorescence measurements by providing a larger cell measurement surface with a higher number of cells in precise microscope focus. Furthermore, the results obtained by the 2D-assembly were validated by FCM measurements of CellROX^®^ stained cells. Similarly to FCM, the results of the DEP assisted 2D-structure characterization were presented as histograms (Figure 5e) demonstrating a shift of the fluorescence profiles of CellROX^®^ Green stained cells between the control and 10^−5^ M Cu at 120 min of exposure (Figure 5f,g), although the shift with FCM was larger. However the sensitivity of the 2D-assembled structures was lower than FCM as demonstrated by the smaller shift in the fluorescent intensity at Cu exposure. This should be expected given that the FCM offers very high sensitivity that is unmatched by fluorescence microscopy detection platforms.

### 3.4. 2D-Assembly Based Sensing of Oxidative Stress during Short-Term Exposure to Contaminants

**Figure 6:**
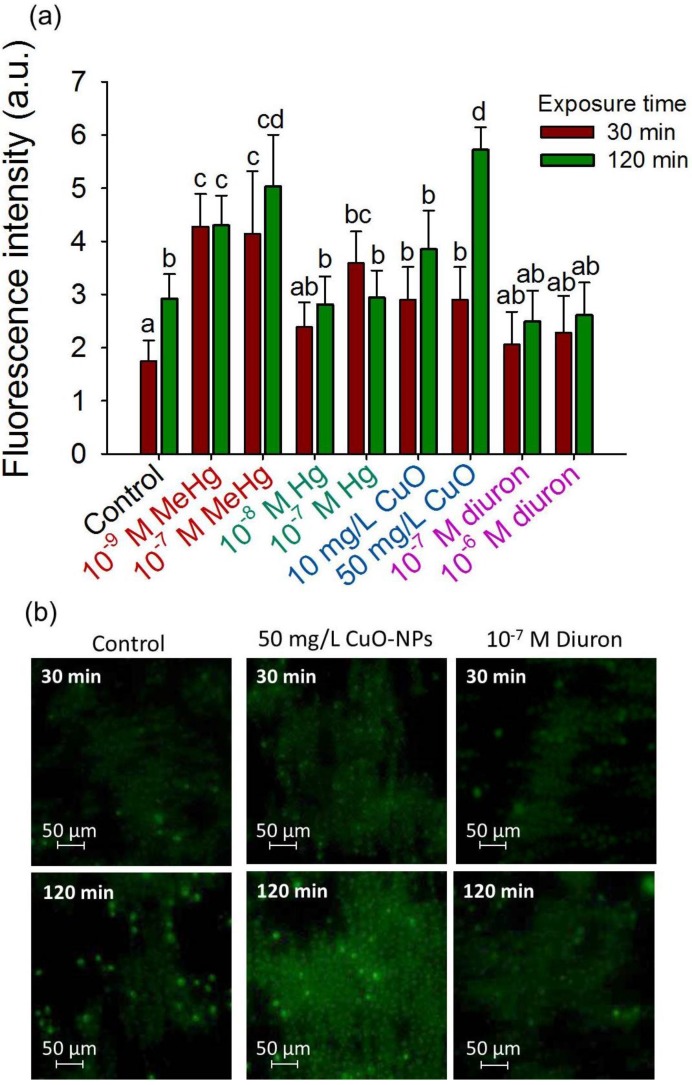
2D-cell array sensing of oxidative stress during short-term exposure to contaminants. (**a**) Mean corrected CellROX^®^ fluorescent intensity (*CF*) for different contaminants; Different letters indicate significant differences between means (*p* < 0.05, Student-Neuman-Keuls test); (**b**) Fluorescence microscopy of the CellROX^®^ stained cells collected in 2D-assembly by positive DEP in the absence of contaminant (control), presence of 50 mg/L CuO-NPs and 10^−7^ M diuron; Exposure time of 30 min and 120 min.

The 2D-assemblies were used to probe the contaminant-induced ROS in *C. reinhardtii* during short-term exposure of 30 and 120 min (Figure 6a–d). Among the tested contaminants, MeHg induced significant production of ROS at concentrations of 10^−9^ and 10^−7^ M where *CF* values increased from 4.28 ± 0.61 and 4.14 ± 1.18 at 30 min to 4.3 ± 0.56 and 5.03 ± 0.98 at 120 min, respectively. No enhancement of cellular ROS was observed in the presence of 10^−8^ M of Hg or diuron. 10^−7^ M Hg however induced a rapid increase on ROS production, as the *CF* value of the exposed cell layers are two times higher at 30 min compared to unexposed controls. Nevertheless, the ROS production level off with exposition time with *CF* of 2.94 ± 0.51 at 120 min, comparable with the one for control 2.92 ± 0.46 at 120 min. Significant increase of ROS production was observed with 10 and 50 mg·L^−1^ of CuO-NPs after 120 min exposure with *CF* of 3.85 ± 0.73 and 5.72 ± 0.43, respectively.

These results are consistent with literature reporting other means of characterizing the effect of CuO-NPs on ROS content in algae [2,40]. CuO-NPs were shown to cause a strong production of ROS in *C. reinhardtii*, bioluminescent bacterial strains of *E. coli* and *P. chlororaphis* with a concentration range in the order of mg·L^−1^ [2,52,53]. The formation of ROS by CuO-NPs was mainly driven by the solubilization of Cu ions than by direct interactions between the nanoparticles and the cells [52,53] and showed to be independent of photosynthetic activity [54]. The lack of oxidative stress and lipid oxidation with diuron were already shown in *Scenedesmus obliquus* [55] and *C. reinhardtii* [40]. Interestingly for 10^−7^ M MeHg or Hg, the rapid increase of the percentage of CellROX^®^ positive cells at 30 min was followed by a leveling off or even a decrease at 120 min. This observation suggests that after 30 min exposure to contaminants *C. reinhardtii* is able to activate the antioxidant defense mechanisms, e.g., CAT, ascorbate peroxidase (APX) and SOD enzymes [56]. Indeed, the activation of these three enzymes was induced by Hg in *C. reinhardtii* [57], but this study also reported a high accumulation of ROS upon Hg exposition comparing to our results [57].

## 4. Conclusions

AC-positive dielectrophoresis makes possible rapid and convenient cell trapping and assembling in compact two-dimensional structures with no alteration of the cellular oxidative status. The efficient DEP-driven cell assembly was obtained at for AC-electric field of 100 V·cm^−1^ and 100 Hz field applied for 30 min between four point stainless steel needle electrodes. The formation of single-layer, DEP-trapped and close-packed layer allows facile and reliable microscopy analysis. Combined with CellROX^®^ oxidative stress probe staining and fluorescence microscopy detection, 2D-assembly were used to follow the *C. reinhardtii* oxidative status during short-term exposure to different environmental contaminants. The results revealed significant enhancement of cellular ROS and oxidative stress when *C. reinhardtii* was exposed to increasing concentrations of MeHg, CuO-NPs and 10^−5^ M Cu. No cellular ROS increase was observed for diuron, Hg and lower Cu concentration exposure. In a combination with different stains, the 2D-assembly could allow the multiplex sensing at different biological end points and can be used as lab-on-a-chip “reactor” for studying the mode of toxic action of different contaminants and reducing the use of biological material, chemical consumable and time.
